# Plasma biomarkers of inflammation, coagulation, and brain injury as predictors of delirium duration in older hospitalized patients

**DOI:** 10.1371/journal.pone.0226412

**Published:** 2019-12-19

**Authors:** J. Brennan McNeil, Christopher G. Hughes, Timothy Girard, Lorraine B. Ware, E. Wesley Ely, Rameela Chandrasekhar, Jin H. Han

**Affiliations:** 1 Division of Allergy, Pulmonary, and Critical Care Medicine, Vanderbilt University Medical Center, Nashville, Tennessee, United States of America; 2 The Critical Illness, Brain Dysfunction and Survivorship (CIBS) Center, Vanderbilt University Medical Center, Nashville, Tennessee, United States of America; 3 Division of Anesthesiology Critical Care Medicine, Vanderbilt University Medical Center, Nashville, Tennessee, United States of America; 4 Clinical Research, Investigation, and Systems Modeling of Acute illness (CRISMA) Center in the Department of Critical Care Medicine, University of Pittsburgh School of Medicine, Pittsburgh, Pennsylvania, United States of America; 5 Veteran Affairs Geriatric Research, Education, and Clinical Center (GRECC), Nashville, Tennessee, United States of America; 6 Department of Biostatistics, Vanderbilt University Medical Center, Nashville, Tennessee, United States of America; 7 Department of Emergency Medicine, Vanderbilt University Medical Center, Nashville, Tennessee, United States of America; University of Florence, ITALY

## Abstract

**Background:**

Delirium's pathophysiology is poorly understood. We sought to determine if plasma biomarkers of inflammation, coagulation, endothelial activation, and blood brain barrier (BBB) injury were associated with emergency department (ED) delirium duration.

**Methods:**

We enrolled hospitalized patients who were 65 years or older from the ED. Plasma biomarkers of inflammation (interleukin-6 [IL-6], IL-8, soluble tumor necrosis factor receptor I [sTNFRI]), coagulation (Protein C), endothelial activation (plasminogen activating inhibitor-1 [PAI-1]), and BBB injury (S100B) at were measured using blood obtained at enrollment. The dependent variable was ED delirium duration which was determined by the Brief Confusion Assessment Method assessed in the ED and hospitalization. Proportional odds logistic regression analyses were performed adjusted for relevant confounders and allowing for interaction by baseline dementia status.

**Results:**

A total of 156 patients were enrolled. IL-6 (POR = 1.59, 95%CI: 1.09–2.32) and PAI-1 (POR = 2.96, 95%CI: 1.48 to 6.85) were independently associated with more prominent ED delirium duration in subjects without dementia only. No significant associations between IL-8, Protein C, sTNRFI, and S100B and ED delirium duration were observed.

**Conclusions:**

Plasma Biomarkers of systemic inflammation and endothelial activation are associated with ED delirium duration in older ED patients without dementia.

## 2. Introduction

Delirium is a form of acute brain failure that affects 10% of older emergency department (ED) patients [1–5] and 25% of hospitalized patients[6–11]. Delirium is not a transient event, commonly lasting 3 to 7 days[12, 13]. Each day a patient is delirious has been associated with an increase in long-term mortality [14] and carries with it worse 6-month functional and cognitive decline[12]. Unfortunately, there are no universally accepted interventions designed to reduce delirium incidence or duration[15]. Our lack of understanding of delirium’s pathogenesis is a significant barrier to optimal delirium management.

Several hypotheses for delirium pathogenesis have been proposed[16] but the neuroinflammatory hypothesis is the most prominent. This hypothesis posits that an acute medical illness causes delirium primarily through systemic and central nervous system (CNS) inflammation. During acute illness, systemic cytokines and proinflammatory mediators are released into the bloodstream and stimulate the vagus nerve, choroid plexus, and circumventricular organs[17]. These events also cause endothelial activation, which leads to activation of coagulation pathways, increased microvascular permeability, and impaired microcirculatory function[18, 19]. This systemic insult can lead to blood-brain barrier (BBB) injury and CNS inflammatory cascade[20–24]. Brain parenchymal cells, such as the microglia, become activated leading to increased expression of proinflammatory mediators in the CNS and ultimately to neuronal injury.

In critically-ill patients, we observed that plasma biomarkers of inflammation, coagulation, endothelial activation, and BBB injury were associated with delirium[25, 26]. Studies in non-critically ill patients have evaluated the relationship between these pathways and delirium, but their findings have been inconsistent [27–30]. In addition, these non-intensive care unit (ICU) studies evaluated delirium as a present-absent event rather than examining its duration, which may be a better discriminator of subsequent adverse outcomes. We, therefore, sought to determine if plasma biomarkers of inflammation (interleukins 6 and 8 [IL-6, IL-8][31, 32], soluble tumor necrosis factor receptor I [sTNFRI])[26], coagulation (Protein C)[26], endothelial activation (plasminogen activating inhibitor-1 [PAI-1])[25], and BBB injury (S100B)[33] were associated with ED delirium duration in older ED patients admitted to the hospital.

## 3. Materials and methods

### Study design and setting

We conducted this prospective cohort study as part of the DELINEATE study evaluating delirium in the ED and its extension into the hospital. This study was conducted at a tertiary care, academic hospital[12]. Results from the DELINEATE study have been previously reported [12], but the hypotheses and findings presented herein are original and have not been previously published. The Vanderbilt institutional review board reviewed and approved this study.

### Selection of participants

Patients were enrolled from the Vanderbilt University Medical Center ED between March 2012 and November 2014. Consecutive enrollment with written, informed consent occurred Monday through Friday at four randomly selected 4-hour blocks per week (8A –12P, 10A – 2P, 12P – 4P, 2P – 6P) for a total of 16 hours per week. Research assistants reviewed the informed consent document with the patient. Written informed consent was obtained from an authorized surrogate if the patient could not state the purpose of the study or name one risk to participating in the study.

Patients were included if they were 65 years or older and in the ED for less than four hours at the time of enrollment. Patients were excluded if they were non-English speaking, previously enrolled, deaf, comatose, non-verbal or unable to follow simple commands prior to their current illness, were considered unsuitable for enrollment by the treating physician or nurse, were unavailable for enrollment within the 4-hour time limit secondary to clinical care (e.g., resuscitations, procedures), or were likely to be discharged home according to the ED. Patients who were non-verbal or unable to follow simple commands prior to their acute illness were considered to have end-stage dementia and were excluded because the delirium assessment tool used in this study was not validated for this patient group. Because only 8–17% of older enrolled ED patients were expected to be delirious[1, 2, 5], all delirious and one out of six randomly selected non-delirious older ED patients were enrolled to maximize the feasibility of our study.

### Sample collection, storage, and biomarker measurement

We collected blood in citrate anti-coagulated collection tubes immediately upon study enrollment. Tubes were placed on ice and centrifuged at 3000g within 1 hour to isolate plasma. Supernatants were stored in aliquots at -80C until batched biomarker analyses were performed in duplicate after prospective enrollment was completed. Sample dilution plates were created to avoid multiple freeze thaw cycles. Electrochemiluminescent assays were performed for IL-6, IL-8, PAI-1, sTRNFI (Meso Scale Discovery; Rockville, MD). Sandwich ELISAs were performed for S100B (Millipore; Billerica, MA) and Protein C (Helena Laboratories; Beaumont, Texas).

### Primary outcome variable

The primary outcome variable was the total number of days a delirious ED patient remained delirious throughout the hospitalization (ED delirium duration). Because the serum biomarkers measured at enrollment are less likely to affect incident delirium especially later in the hospital course, patients who were not delirious in the ED were assigned an ED delirium duration of 0 days even if they later developed delirium during hospitalization. Delirium was assessed by trained research assistants in the ED at the time of enrollment (0 hours), at 3 hours and daily during the hospitalization for 7 consecutive days after the ED visit or until hospital discharge or death, whichever came first. A patient was considered to be delirious in the ED if either the 0- or 3-hour delirium assessment was positive. The delirium episode was considered resolved if the patient was non-delirious for two consecutive days. In non-mechanically ventilated patients, the modified Brief Confusion Assessment Method was used to ascertain delirium. It is 82% to 86% sensitive and 93% to 96% specific for delirium as diagnosed by a psychiatrist with a kappa of 0.87 indicating excellent inter-observer reliability[34]. In mechanically ventilated patients, the Confusion Assessment Method for the Intensive Care Unit (CAM-ICU) was used to ascertain delirium[35]. The CAM-ICU is 93% to 100% sensitive and 98% to 100% specific for delirium in these patients, with a kappa of 0.96 indicating excellent inter-observer reliability[36].

#### Covariates

Medical record review was used to collect dementia status, comorbidity burden, severity of illness, and the presence of a central nervous system diagnosis. A patient was considered to have dementia if they had: (i) documented dementia in the medical record, (ii) a premorbid IQCODE greater than a cut-off of 3.38[37], or (iii) they were prescribed cholinesterase inhibitors prior to admission. The Charlson Comorbidity Index was used to quantify the patient’s comorbid burden[38]. The Acute Physiology Score (APS) of the Acute Physiology and Chronic Health Evaluation II (APACHE II) score was used to quantify severity of illness[39]. For this analysis, age, as scored by the APACHE II, was incorporated into the APS. The presence of a central nervous system (CNS) diagnosis (meningitis, seizure, cerebrovascular accident, intraparenchymal hemorrhage, etc.) was determined by two physician reviewers via medical record review. A third physician reviewer adjudicated any disagreements. Infection diagnoses during the enrollment hospital admission and use of home steroids were collected from the electronic medical record.

### Data analysis

All biomarker measurements were log transformed to minimize the influence of extreme outliers. Proportional odds logistic regression was performed to examine the independent association of biomarkers of inflammation, coagulation, endothelial activation, and BBB injury with ED delirium duration. These models were adjusted for dementia, baseline functional status, comorbidity burden, severity of illness, CNS diagnosis, infection diagnosis, and use of home steroids. These covariates were chosen *a priori* based upon literature review and expert opinion. Since dementia is associated with many of these mechanistic pathways and with delirium, we also evaluated if the association between biomarkers and ED delirium duration was modified by dementia. We allowed for potential interactions between biomarkers and dementia using a separate cross-product interaction term. Because this analysis was exploratory in nature, we considered effect modification to be present if the interaction term’s p-value was less than 0.20. Proportional odds ratios with their 95% confidence intervals (95%CI) were reported. All statistical analyses were performed with SAS version 9.4 (SAS Institute, Carey, NC) and open source R statistical software, version 3.0.2 (http://www.r-project.org/).

## 4. Results

We enrolled and measured biomarker data on 156 patients; 64 were delirious at initial presentation with a median (IQR) ED delirium duration of 3 (2, 5) days. Patient characteristics for delirious and non-delirious patients at initial presentation are compared in **Table 1**. Delirious patients were slightly older and were more likely to be female, non-white, have multiple morbidities, and to have dementia and higher severity of illness than non-delirious patients. Delirious patients were also more likely to have a systemic infection and a CNS diagnoses. However, they were less likely to be on home corticosteroids.

**Table 1:** Patient characteristics and demographics.

	Non-Delirious at Presentation n = 92	Delirious at Presentation n = 64
Median Age (IQR)	72.5 (69, 79)	74 (68, 82)
Female gender	45 (48.9%)	42 (65.6%)
Non-white race	8 (8.7%)	10 (15.6%)
Dementia	23 (25.0%)	48 (75.0%)
Median (IQR) OARS ADL	25 (21, 27)	13 (7, 20)
Median (IQR) IQCODE	3.19 (3.00, 3.56)	4.13 (3.19, 4.69)
Median Charlson (IQR)	3 (1, 4.5)	3 (2, 5)
Median APS with age (IQR)	8 (7, 10.5)	9 (8, 12.5)
Systemic infection diagnosis	25 (27.2%)	33 (51.6%)
CNS diagnosis	10 (10.8%)	12 (18.8%)
Home corticosteroid use	21 (22.8%)	9 (14.1%)
Biomarkers Log_IL-6 (pg/mL) Log_IL-8 (pg/mL) Log sTNRFI (pg/mL) Log Protein C (pg/mL) Log PAI-1 (pg/mL) Log S100B (pg/mL)	0.56 (0.04, 1.63) 1.17 (0.64, 1.68) 8.52 (8.03, 8.86) 4.32 (3.91, 4.57) 12.34 (11.96, 12.90) 2.72 (1.66, 3.33)	1.24 (0.82, 1.96) 1.53 (1.03, 1.94) 8.44 (7.99, 9.00) 4.28 (3.70, 4.57) 12.65 (12.09, 13.09) 3.09 (0.30, 4.00)	

Abbreviations: IQR, Interquartile range; APS, Acute Physiology Score; IL, interleukin; sTNRFI, soluble tumor necrosis factor receptor 1; PAI-1, plasminogen activation inhibitor-1

**Table 2** lists the proportional odds ratios for increased ED delirium duration for each biomarker from the multivariable proportional odds regression models. With regard to systemic inflammation, only IL-6 and IL-8 were significantly associated with ED delirium duration. For IL-6, baseline dementia modified this association (interaction p-value = 0.1826) such that higher concentrations of IL-6 were more prominently associated with longer ED delirium duration in patients without dementia (POR = 1.59, 95%CI: 1.09–2.32).

**Table 2:** Plasma Biomarkers and Their Association emergency department delirium duration.

Biomarker	POR (95%CI)	Biomarker*Dementia P-value
Log IL-6 No Dementia Yes Dementia	1.37(1.05,1.81) 1.59 (1.09, 2.32) 1.14 (0.80, 1.62)	0.1826
Log IL-8	1.57 (1.08, 2.29)	0.2170
Log sTNFRI	1.15 (0.73, 1.83)	0.6967
Log Protein C	1.21 (0.68, 2.13)	0.9519
Log PAI-1 No Dementia Yes Dementia	1.85(1.15,2.99) 2.96 (1.28, 6.85) 1.48 (0.83, 2.62)	0.1786
Log S100B	1.04 (0.82, 1.31)	0.9315

Proportional odds logistic regression examining the effect of plasma biomarkers of inflammation, coagulation, and blood-brain barrier injury on emergency department delirium duration in 156 older emergency department patients. Plasma biomarkers collected at enrollment. Separate models were created for each biomarker. The proportional odds regression models were adjusted for dementia, baseline functional status, comorbidity burden, severity of illness, CNS diagnosis, infection diagnosis, or use of home steroids. Proportional odds ratios (POR) with their 95% Confidence Intervals (95%CI) are reported. If the plasma biomarker*dementia interaction was < .20, then proportion odds ratios (POR) were also reported for patients with and without dementia.

Biomarkers of coagulation as measured by Protein C and BBB injury as measured by S100B were not significantly associated with ED delirium duration. Higher PAI-1, a marker of endothelial activation, however, was significantly associated with prolonged ED delirium duration. This association was modified by baseline dementia (interaction p-value = 0.1786) such that the relationship was more prominent in patients without dementia (POR = 2.96, 95%CI: 1.48 to 6.85).

## 5. Discussion

We observed that plasma IL-6, IL-8, and PAI-1 measured in the ED were significantly associated with prolonged ED delirium durations in patients without dementia admitted to the hospital. Protein C and S100B, which are biomarkers of coagulation and BBB / astrocyte injury, respectively, were not associated with ED delirium duration. These findings suggest that systemic inflammation and endothelial activation, respectively, may be involved in delirium pathophysiology. The impacts of these pathophysiological processes, however, seem to only be apparent in those with intact baseline cognition.

Similar to our findings, several investigations have observed associations between plasma IL-6 and IL-8 and development of delirium in older medical[27, 40] and hip fracture[27, 32, 41] patients. While this suggests that systemic inflammation may play a prominent role in delirium pathogenesis, this association has not been consistently observed[28, 29]. We also did not observe an association between sTNFR1 and ED delirium duration. sTNFR1 is a receptor for Tumor Necrosis Factor-α and has previously been observed to be associated with delirium in ICU cohorts[26, 42]. The abovementioned discordant observations between our study and others may be related to the timing of these biomarker measurements. We collected blood within 4 hours of enrollment in the ED. The inflammatory response to an acute illness is complex and varies from patient to patient throughout the episode of delirium and hospitalization[32]. Such discrepancies may also be secondary to the differences in cohort. As opposed to studies conducted in the ICU, the ED population is markedly more diverse and includes medical, surgical, and neurological patients across the entire spectrum of illness severity. To better elucidate the role of systemic inflammation in the pathogenesis of delirium, future studies should perform serial plasma biomarker measurements in a larger cohort of patients.

We also examined potential downstream effects of systemic inflammation by measuring plasma biomarkers of coagulation, endothelial dysfunction, and BBB injury[18, 20, 21, 43, 44]. Previous investigations in critically patients have observed that plasma Protein C[26], PAI-1[45], and S100B[25, 45, 46] are associated with delirium. In our cohort, however, we only observed PAI-1 to be associated with delirium. Similar to the plasma inflammatory biomarkers, the reasons for these discordant observations may be related to the timing of biomarker measurement and the cohort studied.

One novel aspect of our study is that we observed that the associations between plasma IL-6 and PAI-1 were modified by pre-existing cognition. Surprisingly, the associations between these plasma biomarker measurements and ED delirium duration were most apparent in patients without pre-existing cognitive impairment. The reasons for this are currently unclear. Patients who are cognitively intact at baseline likely require a more noxious insult (e.g., higher systemic inflammation and endothelial dysfunction) to precipitate delirium[47]. Conversely, patients who are more vulnerable to developing delirium, such as those with dementia, may become delirious with even a minor inflammatory insult. These patients may not require a high degree of systemic inflammation and endothelial dysfunction to develop delirium, which may explain the lack of associations between IL-6, IL-8, and PAI-1 and ED delirium duration. Further, patients with dementia may have chronic dysfunction in inflammatory, coagulation, and BBB function which alters their response to acute illness. It is also possible that the pathophysiologic mechanisms for delirium may differ between those with and without pre-existing dementia. Future studies with a larger sample size should confirm these findings.

Our study has several limitations. We only enrolled patients during the daytime hours potentially introducing selection bias; patients who were present in the evening and early morning may have been older, multi-morbid, and sicker. A large number (n = 576) refused to participate in the study, potentially introducing additional selection bias. Patients who refused to participate were slightly older, were more likely to be white, and slightly more likely to reside in a nursing home[12]. Our sample size was relatively small. The absence of an association between sTNRF1, Protein C, and S100B and ED delirium duration may have been a type 2 error (false negative). Inherent to most prospective cohort studies, unmeasured (e.g., drug exposure during hospitalization) and residual confounding may have still existed. In addition, dementia status was characterized by medical record review and IQCODE and its severity was not quantified.

We measured biomarker concentrations at one time point early upon presentation. While this time point provides important prognostic value for the remaining hospital course, biomarker concentrations may differentially change in response to the illness progression and medical therapies. Our sample size was relatively small. The absence of an association between sTNRF1, Protein C, and S100B and ED delirium duration may have been a type 2 error (false negative). Lastly, this was a single center study performed at a tertiary center with medically complex patients who have a wide array of illnesses. Our findings may not be generalizable to other settings.

In conclusion, plasma IL-6, IL-8, and PAI-1 measured in the ED are associated with ED delirium duration in acutely ill older patients, but these associations were significant only in patients without pre-existing dementia. Our data suggest that systemic inflammation and endothelial dysfunction are associated with delirium pathogenesis, but this association may be modified by baseline cognition. Future studies with serial biomarker measurements throughout delirium’s course are needed to better clarify these relationships.

## Supporting information

S1 Table Contains relevant clinical data used in this analysis. (CSV)
